# ICAM‐1 Activates Platelets and Promotes Endothelial Permeability through VE‐Cadherin after Insufficient Radiofrequency Ablation

**DOI:** 10.1002/advs.202002228

**Published:** 2021-01-04

**Authors:** Jian Kong, Changyu Yao, Shuying Dong, Shilun Wu, Yangkai Xu, Ke Li, Liang Ji, Qiang Shen, Qi Zhang, Rui Zhan, Hongtu Cui, Changping Zhou, Haigang Niu, Guoming Li, Wenbing Sun, Lemin Zheng

**Affiliations:** ^1^ Department of Hepatobiliary Surgery Beijing Chaoyang Hospital Capital Medical University Beijing 100043 P. R. China; ^2^ The Institute of Cardiovascular Sciences and Institute of Systems Biomedicine School of Basic Medical Sciences Peking University Health Science Center Key Laboratory of Molecular Cardiovascular Sciences of Ministry of Education Key Laboratory of Cardiovascular Molecular Biology and Regulatory Peptides of Ministry of Health Beijing Key Laboratory of Cardiovascular Receptors Research Beijing 100191 P. R. China; ^3^ Beijing Tiantan Hospital China National Clinical Research Center for Neurological Diseases Advanced Innovation Center for Human Brain Protection Capital Medical University Beijing 100050 P. R. China

**Keywords:** endothelial permeability, hepatocellular carcinoma, ICAM‐1, platelets, radiofrequency ablation

## Abstract

Radiofrequency ablation (RFA) for hepatocellular carcinoma (HCC) often leads to aggressive local recurrence and increased metastasis, and vascular integrity and platelets are implicated in tumor metastasis. However, whether interactions between endothelial cells and platelets induce endothelial permeability in HCC after insufficient RFA remains unclear. Here, significantly increased CD62P‐positive platelets and sP‐selectin in plasma are observed in HCC patients after RFA, and tumor‐associated endothelial cells (TAECs) activate platelets and are susceptible to permeability after heat treatment in the presence of platelets in vitro. In addition, tumors exhibit enhanced vascular permeability after insufficient RFA in mice; heat treatment promotes platelets‐induced endothelial permeability through vascular endothelial (VE)‐cadherin, and ICAM‐1 upregulation in TAECs after heat treatment results in platelet activation and increased endothelial permeability in vitro. Moreover, the binding interaction between upregulated ICAM‐1 and Ezrin downregulates VE‐cadherin expression. Furthermore, platelet depletion or ICAM‐1 inhibition suppresses tumor growth and metastasis after insufficient RFA in an orthotopic tumor mouse model, and vascular permeability decreases in ICAM‐1^−/−^ mouse tumor after insufficient RFA. The findings suggest that ICAM‐1 activates platelets and promotes endothelial permeability in TAECs through VE‐cadherin after insufficient RFA, and anti‐platelet and anti‐ICAM‐1 therapy can be used to prevent progression of HCC after insufficient RFA.

## Graphical abstract

This work demonstrates that platelets are activated in hepatocellular carcinoma patients after radiofrequency ablation (RFA), and tumor‐associated endothelial cells (TAECs) are involved in the process. Intercellular cell adhesion molecule‐1 (ICAM‐1) activates platelets and promotes vascular permeability in TAECs through VE‐cadherin after insufficient RFA. Anti‐platelet and anti‐ICAM‐1 therapy could be used to prevent progression of HCC after insufficient RFA.

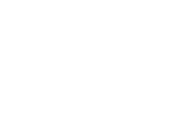

## 1 Introduction

Hepatocellular carcinoma (HCC) is a leading cause of cancer‐related death worldwide, accounting for more than 700 000 deaths per year.^[^ ^1^ ^]^ Liver transplantation, surgical resection, and radiofrequency ablation (RFA) are the first‐line therapies for small HCC.^[^ ^2^ ^]^ Studies have demonstrated that RFA is inferior to surgical resection in terms of local control and disease‐free survival^[^ ^3^, ^4^, ^5^ ^]^ and often leads to aggressive local recurrence and increased metastasis.^[^ ^6^, ^7^, ^8^ ^]^ However, the underlying mechanisms by which insufficient RFA significantly promotes tumor progression remain unclear.

Accumulating evidence indicates that vascular integrity and platelets are implicated in cancer metastasis. Vascular integrity is maintained by endothelial cell‐to‐cell junctions, which are composed of the adherens junction complex and tight junction complex.^[^ ^9^, ^10^ ^]^ Loss of adhesion molecules damages vascular integrity and increases vascular permeability, thereby facilitating tumor growth and metastasis,^[^ ^11^, ^12^ ^]^ and VE‐cadherin, an adherens junction complex protein,^[^ ^13^, ^14^ ^]^ regulates tumor vascular permeability and metastasis.^[^ ^15^, ^16^, ^17^ ^]^ In addition, platelets play important roles in inflammation, tumor growth, angiogenesis, and metastasis, as well as coagulation and hemostasis,^[^ ^18^, ^19^, ^20^ ^]^ and assist tumor cells to intravasate, escape immune surveillance, and extravasate.^[^ ^21^, ^22^ ^]^ During intravasation, the platelet‐tumor cell interaction enhances angiogenesis and leakage of blood vessels, induces tumor epithelial‐mesenchymal transition, and facilitates tumor cell invasion into vessels.^[^ ^23^, ^24^ ^]^ Nucleotides derived from platelets enhance the transendothelial migration and metastasis of tumor cells via P2Y2 receptor.^[^ ^25^ ^]^ Notably, intercellular adhesion molecule 1 (ICAM‐1), a transmembrane glycoprotein that belongs to the immunoglobulin‐like superfamily, is expressed in various cell types, including endothelial cells, leukocytes, and tumor cells,^[^ ^26^, ^27^ ^]^ and is involved in inflammation, tumor growth, and metastasis, and coronary artery disease.^[^ ^28^, ^29^, ^30^ ^]^ Indeed, firm adhesion between endothelial cells and platelets is mediated by platelet *α*_IIb_ *β*_3_ with endothelial *α*_V_ *β*_2_ and ICAM‐1 and leads to platelet activation.^[^ ^31^ ^]^ These findings suggest that the platelet–endothelial cell interaction is implicated in endothelial permeability and tumor intravasation into vessels. However, no studies have investigated whether insufficient RFA affects endothelial permeability and the interaction between endothelial cells and platelets in HCC.

This present study aimed to determine whether interactions between endothelial cells and platelets induce endothelial permeability in HCC after insufficient RFA. Our findings suggest that ICAM‐1 activates platelets and facilitates endothelial permeability after insufficient RFA, and anti‐platelet and anti ICAM‐1 therapy could be used to prevent HCC progression after RFA.

## 2 Results

### 2.1 TAECs Are Involved in Platelet Activation after Insufficient RFA

**Figure 1:**
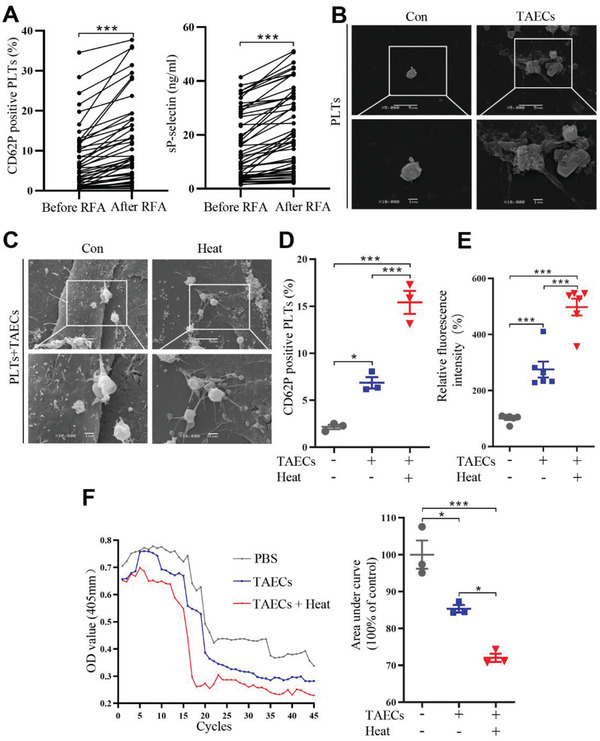
Platelet activation in HCC patients after RFA and platelet adhesion and aggregation induced by TAECs. A) RFA is performed in 53 HCC patients, and CD62P‐positive platelets and sP‐selectin in plasma before and after RFA are evaluated (*n* = 53, ***p < 0.001 by paired *t*‐test). B) SEM showing the morphology of platelets after incubation with or without TAECs. Scale bar = 5 µm. C) SEM showing the morphology of platelets after incubation with TAECs with or without heat treatment. Scale bar = 5 µm. D) Platelets are cocultured with or without TAECs, which are treated with or without heat. Platelets are collected and CD62P‐positive platelets are measured using flow cytometry (*n* = 3, *p < 0.05, ***p < 0.001 by one‐way ANOVA). E) TAECs are treated with or without heat, and human platelets are added. The adhered platelets are measured (*n* = 6, ***p < 0.001 by one‐way ANOVA). F) Human platelets are incubated with TAECs with or without heat treatment, and platelet aggregation is measured. The area under the platelet aggregation curve is analyzed (*n* = 3, *p < 0.05, ***p < 0.001 by one‐way ANOVA). PLTs: platelets. All results are expressed as means ± SEM.

Baseline characteristics of 53 included HCC patients are shown in **Table** **1**. Blood samples were collected before RFA and 24 h after RFA. A significant increase in CD62P‐positive platelets was observed in HCC patients after RFA compared with before RFA (**Figure** **1**A), indicating that platelets were activated after RFA. The isolation process did not activate platelets and the representative dot plots of flow cytometry were shown (Figure S1A,B, Supporting Information). Plasma sP‐selectin levels, a marker of platelet activation, were significantly higher in HCC patients after RFA than before RFA (Figure 1A). Meanwhile, high CD62P positive platelet in HCC patients before RFA or after RFA was correlated with poor disease‐free survival, but the differences were not significant (Figure S2A,B, Supporting Information). In the absence of stimulation, platelets showed concave, oval, or disk shape and few pseudopodia. However, after coculture with tumor‐associated endothelial cells (TAECs), platelets aggregated and showed more pseudopodia, and this effect was further enhanced after TAECs were heat‐treated to simulate insufficient RFA in vitro (Figure 1B,C). Moreover, after platelets and TAECs were cocultured, platelets showed a marked increase in surface expression of CD62P, which was further significantly enhanced after heat treatment (Figure 1D). TAECs with heat treatment could adhere to more platelets than those without heat treatment (Figure 1E). Platelet aggregation occurred in the presence of TAECs and was strengthened after the treatment of TAECs with heat (Figure 1F).

**Table 1:** Baseline characteristics and clinical data of patients before RFA

Variables	Patients with RFA (*n* = 53)
Age, *y*	58.0 ± 9.3
Gender (male/female)	47/6
Etiology (negative/HBV/HCV)	3/49/1
AFP [ng mL^−1^]	
≤400	42
>400	11
Cirrhosis (yes/no)	39/14
Child–Pugh A/B/C	22/31/0
Tumor number (single/multiple)	43/10
Tumor size [cm]	
Mean	3.6 ± 0.8
≤3 (n)	47
3‐5 (n)	18
TBIL [µmol L^−1^]	24.1 ± 8.7
ALT [U L^−1^]	27.7 ± 23.0
ALB [U L^−1^]	37.8 ± 5.2
WBC (×10^9^)	3.9 ± 1.7
PLT (×10^9^)	89.0 ± 46.5

### 2.2 Insufficient RFA Enhances Endothelial Permeability of HCC In Vitro and In Vivo

**Figure 2:**
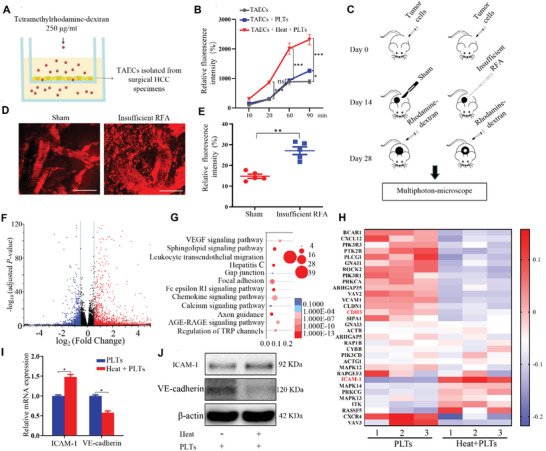
Insufficient RFA and platelets increase the permeability of endothelial monolayers in vitro and in vivo. A) TAECs are cultured on the collagen‐coated transwell inserts and treated with or without heat treatment or platelets. Media containing tetramelthyrhodamine‐dextran (250 µg mL^−1^) are added to the upper chamber. B) Media from the lower chamber are taken to measure fluorescence intensities at 10, 30, 60, and 90 min (*n* = 3, *p < 0.05, ***p < 0.001 by one‐way ANOVA). C) An ectopic tumor model is established using nude mice, and an insufficient RFA is performed. After two weeks, a multiphoton microscope is used to evaluate tumor vascular permeability after intravenous injection of tetramelthyrhodamine‐dextran. D) Representative images of tumor vascular permeability. Scale bar = 100 µm. E) Quantification of relative fluorescence in the sham and insufficient RFA groups (*n* = 5, **p < 0.01 by unpaired *t*‐test). F) Volcano plot indicates the distribution of differentially expressed genes in TAECs with or without heat treatment, which are cocultured with platelets (*n* = 3, Down: *p* < 0.05 and log FC <−0.58; Up: *p* < 0.05 and logFC > 0.58). G) Functional enrichment analysis of differentially expressed genes in TAECs cocultured with platelet after treatment with or without heat is performed using KEGG pathways. H) Representative heat map of differentially expressed genes in TAECs after incubation with platelets with or without heat treatment. Each row represents a gene, and each column represents a group of TAECs. I,J) ICAM‐1 and VE‐cadherin relative mRNA and protein levels in TAECs pretreated with platelets with or without heat treatment (*n* = 3, *p < 0.05 by unpaired *t*‐test). PLTs: platelets. All results are expressed as means ± SEM.

To explore the endothelial permeability of HCC after insufficient RFA, the endothelial permeability assay was performed to measure the traversing of tetramethylrhodamine‐labeled dextran through the monolayer of TAECs pretreated with platelets and/or heat. Pretreatment with platelets allowed more dextran to traverse through the endothelial monolayer; much more dextran traversed through the endothelial monolayer in the presence of platelets and heat treatment than in the presence of only platelets (**Figure** **2**A,B). We further investigated vascular permeability in HCC with or without insufficient RFA in vivo using tetramethylrhodamine‐labeled dextran with a multiphoton microscope (Figure 2C). Markedly more dextran permeated the extravascular space in HCC after insufficient RFA than after sham control treatment (Figure 2D,E).

Heat‐treated TAECs showed increased permeability after interacting with platelets compared with non‐heat‐treated TAECs (Figure 2B). We next investigated the potential mechanisms. Using Kyoto Encyclopedia of Genes and Genomes (KEGG) pathway enrichment analysis of RNA‐seq data, we found that the transendothelial migration pathway was one of the most substantially affected pathways in heat‐treated TAECs after interacting with platelets (Figure 2F,G). In this study, mRNAs with fold changes ≥ 1.5 and *p* values <0.05 were identified as differentially expressed mRNAs (Figure 2H). ICAM‐1, which is involved in the interaction between TAECs and platelets, was one of the markedly upregulated genes, and mRNA and protein expressions of ICAM‐1 were upregulated in heat‐treated TAECs after interacting with platelets (Figure 2I,J). In addition, CDH5 (VE‐cadherin) was one of the significantly downregulated genes in heat‐treated TAECs after interacting with platelets, although the fold change of VE‐cadherin was less than 1.5; mRNA and protein expressions of VE‐cadherin were also downregulated in TAECs after heat treatment interaction with platelets (Figure 2I,J). Moreover, we separated TAECs from tumor tissues of insufficient RFA‐treated mice and sham operation‐treated mice, and found that ICAM‐1 expressions in TAECs from insufficient RFA‐treated mice were increased compared with those from sham operation‐treated mice (Figure S3, Supporting Information). Furthermore, we collected five pairs of tumors before and after RFA and determined expressions of ICAM‐1 in TAECs. The expressions of ICAM‐1 in endothelial cells were increased in tumors after RFA compared with before RFA (Figure S4, Supporting Information).

### 2.3 Heat Treatment Promotes Platelets‐Induced Endothelial Cell Permeability through VE‐Cadherin

**Figure 3:**
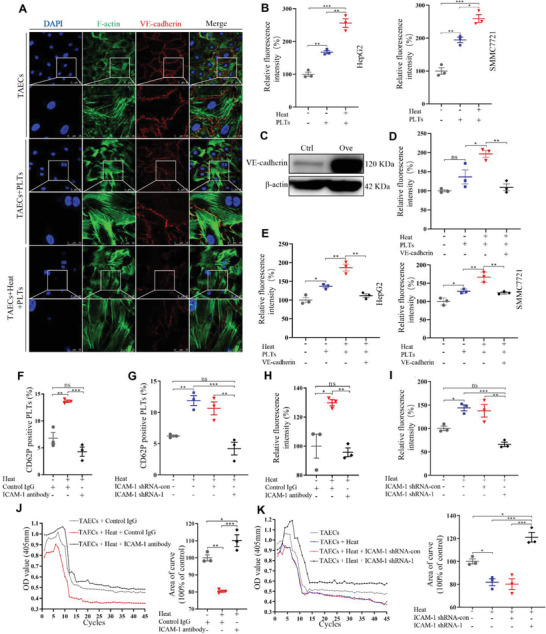
Platelets and heat treatment induce the permeability of endothelial monolayers and facilitate the transendothelial invasion of HCC cells via VE‐cadherin. A) TAEC monolayers are treated with heat and platelets, and VE‐cadherin expressions are examined by laser scanning confocal. Scale bar = 50 µm. B) HepG2 and SMMC7721 cells are used to perform tumor transendothelial assay. The number of HepG2 and SMMC7721 cells invading through the TAEC monolayers is calculated (*n* = 3, *p < 0.05, **p < 0.01, ***p < 0.001 by one‐way ANOVA). C) VE‐cadherin is overexpressed in TAECs using lentivirus and confirmed by western blotting. D) After VE‐cadherin overexpression in TAECs, TAEC monolayers are treated with heat and platelets, and media containing teletstetramelthyrhodamine‐dextran (250 µg mL^−1^) are added to the upper chamber. Media from the lower chamber are taken to measure fluorescence intensities at 90 min (*n* = 3, *p < 0.05, **p < 0.01 by one‐way ANOVA). E) After VE‐cadherin overexpression in TAECs, TAEC monolayers are treated with heat and platelets, and the number of HepG2 and SMMC7721 cells invading through the TAEC monolayers is assessed (*n* = 3, *p < 0.05, **p < 0.01 by one‐way ANOVA). F,G) Anti‐ICAM‐1 antibody and ICAM‐1 shRNA are used to block ICAM‐1 in TAECs. Cocultured platelets are collected, and CD62P‐positive platelets are measured with flow cytometry (*n* = 3, **p < 0.01, ***p < 0.001 by one‐way ANOVA). H,I) Platelet adhesion is examined (*n* = 3, *p < 0.05, **p < 0.01, ***p < 0.001 by one‐way ANOVA). J,K) Platelet aggregation is examined (*n* = 3, *p < 0.05, **p < 0.01, ***p < 0.001 by one‐way ANOVA). PLTs: platelets. All results are expressed as means ± SEM.

To investigate the role of platelets and insufficient RFA in HCC metastasis, we measured tumor endothelial cell interaction and permeability. Platelets induced downregulation of VE‐cadherin in the TAEC monolayer, and heat treatment enhanced this effect (**Figure** **3**A and Figure S5A, Supporting Information), indicating that platelets and heat treatment may influence endothelial cell permeability through VE‐cadherin. Tumor transendothelial assays demonstrated that more HepG2 or SMMC7721 cells migrated through the monolayer of TAECs pretreated with platelets than through that pretreated with the control, and heat treatment enhanced the effect of platelets (Figure 3B). We further demonstrated that the enhanced tetramethylrhodamine‐labeled dextran permeability and tumor cell transendothelial migration induced by treatment of TAECs with platelets and/or heat treatment were attenuated by VE‐cadherin overexpression (Figure 3C–E).

### 2.4 ICAM‐1 Upregulation Results in Platelet Activation and Induces Endothelial Permeability in TAECs after Heat Treatment

ICAM‐1 expression was upregulated in TAECs after heat treatment, and platelets could also upregulate ICAM‐1 expression (Figure S5B, Supporting Information). We next knocked down the expression of ICAM‐1 in TAECs to observe the effect of ICAM‐1 on platelet activation, adhesion, and aggregation (Figure S6A, Supporting Information). Blocking ICAM‐1 using anti‐ICAM‐1 antibody attenuated the effect of heat‐treated TAECs on increased CD62P expressions in platelets (Figure 3F), and ICAM‐1 knockdown using shRNA also attenuated heat‐treated TAECs‐induced increased CD62P expressions in platelets (Figure 3G). TAECs could adhere to more platelets after heat treatment, and anti‐ICAM‐1 antibody and ICAM‐1 shRNA attenuated these effects (Figure 3H,I). Furthermore, anti‐ICAM‐1 antibody and ICAM‐1 shRNA attenuated heat‐treated TAECs‐induced increased platelet aggregation (Figure 3J,K). These results indicate that ICAM‐1 upregulation results in platelet activation in TAECs after heat treatment.

**Figure 4:**
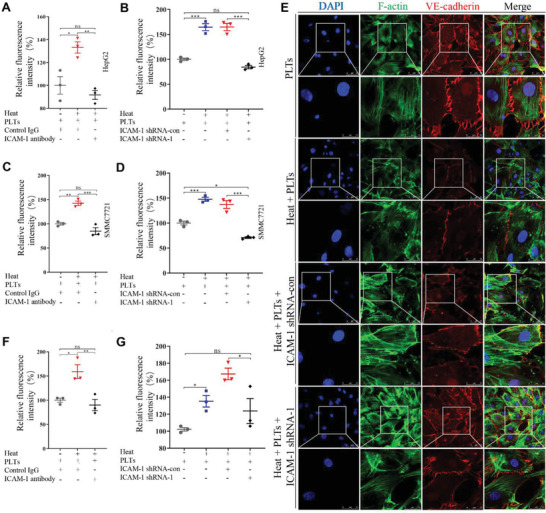
ICAM‐1 up‐regulation in TAECs after heat treatment increases endothelial permeability and facilitates the transendothelial invasion in HCC cells. A,B) The number of HepG2 cells invading the TAEC monolayer is evaluated when anti‐ICAM‐1 antibody is used to block A) ICAM‐1 or B) ICAM‐1 shRNA is used to knock down ICAM‐1 in TAECs (*n* = 3, *p < 0.05, **p < 0.01, ***p < 0.001 by one‐way ANOVA). C,D) The number of SMMC7721 cells invading the TAEC monolayer is evaluated when anti‐ICAM‐1 antibody is used to block C) ICAM‐1 or D) ICAM‐1 shRNA is used to knock down ICAM‐1 in TAECs (*n* = 3, *p < 0.05, **p < 0.01, ***p < 0.001 by one‐way ANOVA). E) VE‐cadherin expressions in TAEC monolayer are examined by a laser scanning confocal microscope when ICAM‐1 shRNA is used to knock down ICAM‐1 expression in TAECs. Scale bar = 50 µm. F,G) Tetramelthyrhodamine‐dextran is used to evaluate endothelial permeability when F) anti‐ICAM‐1 antibody or G) ICAM‐1 shRNA is used in TAECs (*n* = 3, *p < 0.05, **p < 0.01 by one‐way ANOVA). PLTs: platelets. All results are expressed as means ± SEM.

Transendothelial migration assay revealed that more HepG2 or SMMC7721 cells migrated through heat‐treated TAEC monolayers than through non‐heat‐treated TAEC monolayers, while anti‐ICAM‐1 antibody and ICAM‐1 shRNA attenuated these effects (**Figure** **4**A–D). ICAM‐1 shRNA and anti‐ICAM‐1 antibody also attenuated heat treatment‐induced VE‐cadherin downregulation and endothelial cell gaps in TAECs (Figure 4E and Figure S6B,C, Supporting Information). Furthermore, the endothelial permeability assay showed that anti‐ICAM‐1 antibody and ICAM‐1 shRNA could also attenuate heat treatment‐induced increased endothelial cell permeability (Figure 4F,G). Moreover, overexpression of ICAM‐1 in TAECs enhanced the endothelial permeability (Figure S6D, Supporting Information). Taken together, our findings suggest that ICAM‐1 upregulation in TAECs induces endothelial permeability.

### 2.5 Ezrin May Be Involved in ICAM‐1‐Mediated VE‐Cadherin Expression

**Figure 5:**
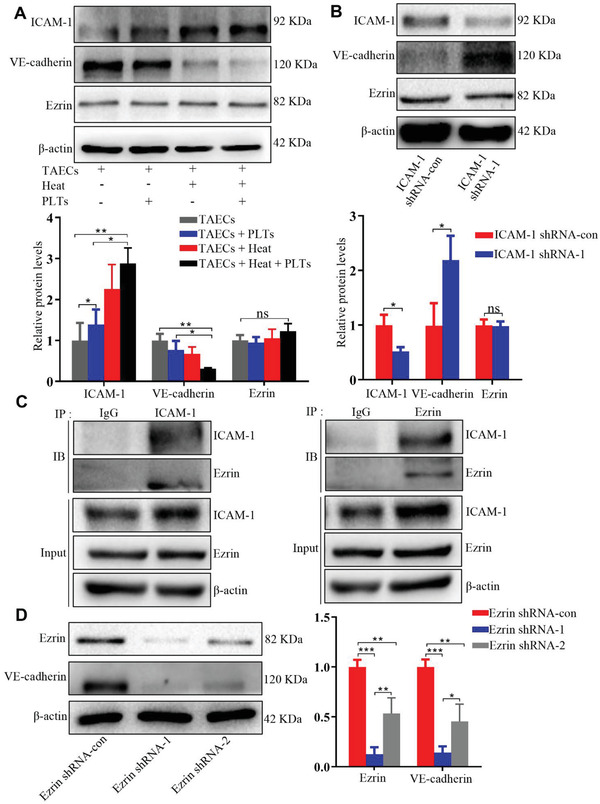
Ezrin may be involved in ICAM‐1‐mediated VE‐cadherin expression. A) Western blotting to measure expressions of ICAM‐1, VE‐cadherin, and Ezrin in TAECs after heat or/and platelet treatments. Protein amount is quantified using ImageJ (*n* = 3, *p < 0.05, **p < 0.01 by one‐way ANOVA). B) Western blotting to measure expressions of ICAM‐1, VE‐cadherin, and Ezrin in TAECs when ICAM‐1 is knocked down. The protein amount is quantified using ImageJ (*n* = 3, *p < 0.05 by Mann‐Whitney test). C) Interaction of endogenous ICAM‐1 with Ezrin is determined by co‐immunoprecipitation analyses. D) Western blotting to measure expressions of VE‐cadherin in TAECs transfected with indicated siRNAs. Protein amount is quantified using ImageJ (*n* = 3, *p < 0.05, **p < 0.01, ***p < 0.001 by one‐way ANOVA). PLTs: platelets. All results are expressed as means ± SEM.

Ezrin is one of ezrin‐radixin‐moesin (ERM) proteins, which generally crosslink between cortical actin filaments and plasma membranes.^[^ ^32^ ^]^ Activation of ERM proteins in endothelial cells could induce cytoskeleton reorganization and increase vascular permeability.^[^ ^33^ ^]^ We investigated the underlying molecular mechanisms by which ICAM‐1 regulates endothelial permeability and examined whether ICAM‐1 affects Ezrin expression in TAECs after heat treatment. Increased ICAM‐1 expression after heat treatment‐induced VE‐cadherin downregulation but had no effect on Ezrin expression (**Figure** **5**A), and ICAM‐1 knockdown increased VE‐cadherin expression but did not affect Ezrin expression in TAECs (Figure 5B). Co‐IP experiments revealed the binding interaction between expressed ICAM‐1 and endogenous Ezrin (Figure 5C). Inhibition of Ezrin resulted in downregulated VE‐cadherin expression in TAECs (Figure 5D), and ICAM‐1 overexpression increased the binding interaction between ICAM‐1 and Ezrin but decreased the bonding interaction between Ezrin and VE‐cadherin (Figure S7A,B, Supporting Information). These results suggest that the binding interaction between upregulated ICAM‐1 and Ezrin may be involved in the downregulation of VE‐cadherin expression.

### 2.6 Platelet Depletion or ICAM‐1 Inhibition Suppresses Tumor Growth and Metastasis after Insufficient RFA

**Figure 6:**
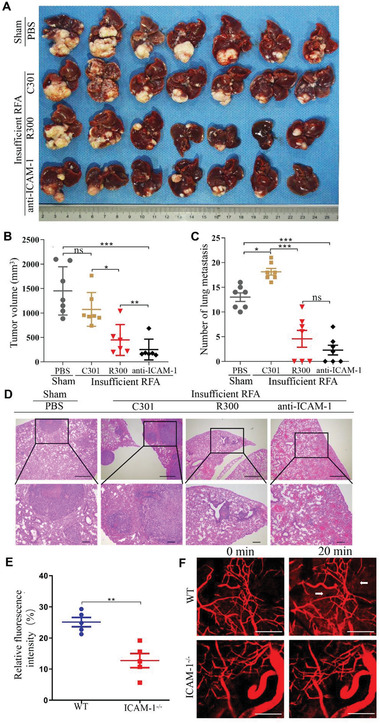
Platelet deletion or ICAM‐1 inhibition suppresses tumor growth and metastasis after insufficient RFA. An orthotopic HCC mouse model is established, and insufficient RFA is performed. A,B) Mice are treated with PBS, C301 (isotype control of R300), R300 (platelet deletion), and anti‐ICAM‐1 antibody (blocking ICAM‐1). Tumor volume is evaluated six weeks after insufficient RFA (*n* = 7, *p < 0.05, **p < 0.01, ***p < 0.001 by one‐way ANOVA). C,D) Lung metastases are examined in mice treated with PBS, C301, R300, and anti‐ICAM‐1 antibody by H&E staining (*n* = 7, *p < 0.05, ***p < 0.001 by one‐way ANOVA). E) An ectopic tumor model is established using wide‐type mice and ICAM‐1^−/−^ mice, and insufficient RFA is performed. After two weeks, mice are intravenously injected with tetramelthyrhodamine‐dextran, and a multi photon‐microscope is used to observe in vivo vascular permeability. Quantification of relative fluorescence in wild‐type mice and ICAM‐1^−/−^ mice (*n* = 5, **p < 0.01 by unpaired *t*‐test). F) Representative images of tumor vascular permeability. White arrows represent leakage of dextran from the vessels. Scale bar = 100 µm. All results are expressed as means ± SEM.

**Figure 7:**
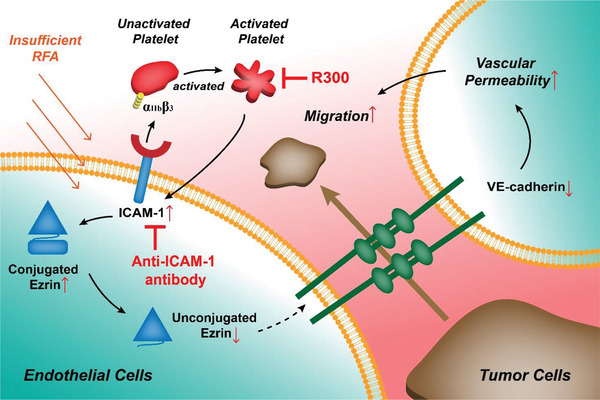
Schematic showing how ICAM‐1 activated by platelets increases endothelial permeability after insufficient RFA. ICAM‐1 in TAECs after insufficient RFA induces platelet aggregation and activation, increases endothelial permeability through Ezrin/VE‐cadherin, and facilitates tumor transendothelial migration. R300 is used for platelet deletion and anti‐ICAM‐1 antibody is used for blockade of ICAM‐1 for potential therapy.

An orthotopic HCC mouse model was further established. Mice were divided into the following four groups: sham + PBS group, insufficient RFA + C301 group, insufficient RFA + R300 group, and insufficient RFA + anti‐ICAM‐1 antibody group (Figure S8A, Supporting Information). The platelet count of mice was assessed to ensure the establishment of long‐term thrombocytopenia during the R300 application (Figure S8B, Supporting Information). There was no difference in body weight among the four groups during the treatment (Figure S8C, Supporting Information), and no abnormality was found in the heart, spleen, and kidney, indicating that these reagents had no side effects in these mice (Figure S8D, Supporting Information). Tumor size did not significantly differ between sham + PBS and insufficient RFA + C301 (a negative control anti‐platelet antibody) groups (**Figure** **6**A,B). However, treatment with anti‐platelet antibody R300 markedly slowed tumor growth compared with that with C301 after insufficient RFA, and treatment with ICAM‐1 antibody could also slow tumor growth (Figure 6A,B). The number of metastatic pulmonary nodules was higher in the insufficient RFA + C301 group than in the sham +PBS group, indicating that insufficient RFA could promote tumor metastasis (Figure 6C). Treatment with anti‐platelet antibody R300 or anti‐ICAM‐1 antibody markedly decreased pulmonary metastasis (assessed by metastatic lesions under microscopy using HE staining) compared with that with C301 after insufficient RFA (Figure 6C,D). We further investigated vascular permeability in HCC after insufficient RFA using ICAM‐1^−/−^ mice and wild‐type mice. Ectopic tumors after insufficient RFA showed more serious vascular permeability in wild‐type mice than in ICAM‐1^−/−^ mice (Figure 6E,F). Taken together, these data suggest that depleting platelet or inhibiting ICAM‐1 suppresses tumor growth and metastasis after insufficient RFA, and ICAM‐1 activates platelets and leads to increased endothelial permeability after insufficient RFA (**Figure** **7**).

## 3 Discussion

In this study, we found that ICAM‐1 in TAECs activated platelets and increased endothelial permeability after insufficient RFA. The findings may provide a new strategy to prevent HCC growth and metastasis after RFA.

RFA is used for tumor ablation, but it remains unclear whether RFA influences the coagulation system or platelet function. A previous study reported that RFA of atrial fibrillation could induce platelet activation,^[^ ^34^, ^35^ ^]^ and the possible mechanisms involve radiofrequency‐induced injury of the subendocardial left atrial wall, which activates the coagulation cascade and inflammatory reaction. Similarly, RFA could induce endothelial cell injury through the thermal effect. In this study, we demonstrated that RFA induced platelet activation in HCC patients. Platelet activation is a risk factor for poor postoperative prognosis in HCC,^[^ ^36^ ^]^ and anti‐platelet agents (aspirin and clopidogrel) have been shown to reduce the risk of HCC and improve the recurrence rate and overall survival in patients with hepatitis B virus‐related HCC after five years following liver resection.^[^ ^37^ ^]^ R300 is an *α*GPIb immunoglobulin G of platelet, which could induce platelet deletion. A previous study showed that R300 could suppress tumor growth and metastasis by inducing platelet deletion.^[^ ^38^ ^]^ Our results showed that R300 markedly slowed tumor growth and metastasis in HCC after insufficient RFA. However, the R300 is currently used in experimental animal models, and relevant clinical researches may be performed in the future. In addition, tumor‐induced platelet activation facilitates intravasation, extravasation, and colonization of secondary sites^[^ ^39^ ^]^ and results in the formation of pseudopodia and extension into lamellipodia and filipodia.^[^ ^40^ ^]^ We found that TAECs promoted platelets to form pseudopodia and undergo aggregation and adhesion, and heat treatment aggravated the process. These results suggest that the TAEC‐platelet crosstalk might be involved in tumor metastasis.

Platelets interact with endothelial cells through surface adhesion molecules or influence endothelial cells through released vesicles, further regulating the endothelial barrier.^[^ ^41^ ^]^ Traversion of tumor cells through the vascular barrier is a crucial step in metastasis, and increased vascular permeability facilitates tumor metastasis. The traditional view is that platelets search for endothelial damage and prevent loss of vascular integrity. However, our results showed that platelets weakened the endothelial barrier, and heat treatment enhanced the effect in vitro. Our findings are partly consistent with those in a previous study, which revealed that platelets amplified and maintained vascular permeability in inflamed joints.^[^ ^42^ ^]^ These findings further suggest that platelets have a double‐edged sword effect on vascular permeability.

VE‐cadherin is an adherens junction protein that maintains vascular integrity, and its downregulation destroys the barrier function of endothelial monolayers.^[^ ^15^, ^43^ ^]^ We found that VE‐cadherin expression was downregulated in TAECs after treatment with platelets and heat, indicating that the endothelial cell barrier was destroyed. Moreover, endothelial monolayers showed increased permeability after interaction with platelets, and heat treatment aggravated this process. Furthermore, VE‐cadherin overexpression reversed increased endothelial permeability. These results indicate that VE‐cadherin plays a crucial role in platelets and heat treatment‐induced vascular permeability.

ICAM‐1, a primary endothelial cell adhesion molecule, interacts with platelet *α*_IIb_ *β*_3_ through endothelial GPIb‐IX‐V and mediates platelet adhesion.^[^ ^31^ ^]^ Studies have indicated that monascus adlay and monacolin K attenuate arterial thrombosis in rats through inhibition of ICAM‐1 in endothelial cells;^[^ ^44^ ^]^ ICAM‐1‐targeted thrombomodulin mitigates tissue factor‐driven inflammatory thrombosis,^[^ ^45^ ^]^ and endotoxemia‐augmented murine venous thrombosis formation is dependent on ICAM‐1 and TLR‐4.^[^ ^46^ ^]^ Consistently, we found that ICAM‐1 expression was upregulated in TAECs after heat treatment, and increased ICAM‐1 expression induced platelet adhesion, aggregation, and activation; ICAM‐1 inhibition or blockade reversed the effects. Moreover, platelet depletion and ICAM‐1 knockout inhibited tumor metastasis and vascular permeability in mice after insufficient RFA. These results suggest that platelet activation induced by ICAM‐1 in endothelial cells is involved in vascular permeability, and ICAM‐1 upregulation after insufficient RFA directly leads to endothelial monolayer barrier injury and promotes transmigration of tumor cells. Another study demonstrated that anti‐ICAM‐1 antibody could be used for myeloma therapy and showed potential anti‐myeloma activity.^[^ ^47^ ^]^ Our results also demonstrated that ICAM‐1 antibody or ICAM‐1 inhibition could inhibit the tumor metastasis in HCC after RFA. Therefore, ICAM‐1 targeted therapy may be used to prevent tumor progression in the future.

ERM proteins, including Ezrin, radixin, and moesin, which are cytoskeleton‐membrane linkers, induce cytoskeleton reorganization in stress fibers, thereby leading to disassembly of focal adhesions and formation of paracellular gaps, which result in increased vascular permeability.^[^ ^33^ ^]^ Ezrin mediates attachment of the intracellular tail of ICAM‐1 to the actin cytoskeleton. Our findings showed that ICAM‐1 interacted with Ezrin, and Ezrin inhibition induced VE‐cadherin downregulation. Our findings are partly consistent with those in a previous study, which showed that MYADM controlled endothelial barrier function through ERM‐dependent regulation of ICAM‐1 expression.^[^ ^48^ ^]^ These findings suggest that increased expressions of ICAM‐1 in TAECs after heat treatment might interact with Ezrin and result in the destruction of the endothelial barrier, and ICAM‐1 interaction with Ezrin may promote vascular permeability.

In conclusion, we found that platelets were activated in HCC patients after RFA, and TAECs were involved in the process. Our findings suggest that ICAM‐1 activates platelets and promotes vascular permeability in TAECs through VE‐cadherin after insufficient RFA, and anti‐platelet and anti‐ICAM‐1 therapy could be used to prevent the progression of HCC after insufficient RFA.

## 4 Experimental Section

##### Patient Selection

This study recruited a total of 53 patients with HCC based on the Milan Criteria and without other severe organ diseases, anti‐platelet drug application, chemotherapy, or interventional therapy in the hospital between September 2012 and August 2014. The Milan criteria were defined as one tumor ≤5 cm or two or three tumors with each tumor ≤3 cm without any vascular invasion or metastasis observed by computed tomography (CT). The study was approved by the ethical review board, and written informed consent was obtained from each patient.

##### CT‐Guided RFA

All surgeries were performed by the same doctors. RFA procedures were performed using Cool‐tip ACT 2030 or ACT 1530 electrodes and an RF generator (RITA 1500; RITA Medical Systems Inc., Manchester, GA, USA, or Covidien Healthcare, Ireland) according to the manufacturers’ protocols. After general anesthesia, the skin entrance point of the RF probe was chosen in the CT scanning plane containing the tumor. With CT monitoring, the RF probe was inserted through the chest wall to the liver until reaching the tumor. After the position of the probe was confirmed as appropriate, RF procedures and strategies were performed.

##### Blood Samples

Written informed consent was obtained from patients, and the study protocol was approved by Beijing Chaoyang Hospital Ethics Committee. Blood samples (10 mL) were collected from patients before RFA and 24 h after RFA. Blood was drawn directly into plastic tubes containing 3.8% buffered sodium citrate and PGI2 was used to avoid platelet activation.

##### Platelet Isolation

Platelets were obtained from the platelet‐rich plasma (PRP). Briefly, 1 mL of blood sample was centrifuged at 200 g for 10 min at room temperature to obtain PRP. Subsequently, platelets were isolated from PRP by centrifugation at 700 g for 10 min. Platelets were resuspended and washed in modified Tyrode's‐HEPES buffer (134 mM NaCl, 2.9 mM KCl, 0.34 mM Na_2_HPO_4_, 12 mM NaHCO_3_, 1 mM MgCl_2_, 20 mM HEPES, and 5 mM glucose; pH 7.3).

##### Flow Cytometry

Isolated platelet was fixed in 4% paraformaldehyde for 20 min, incubated with FITC‐conjugated CD62P‐specific monoclonal antibody (clone #9E1) and isotype control antibody (clone #11711) (R&D Systems, Minneapolis, MN, USA) for 20 min. Subsequently, the platelet was preselected according to the SSC/FSC. A gate was set around the platelets and the activated platelets were analyzed using a Becton Dickinson FACScan to measure the platelet surface expression of CD62P.

##### ELISA

The sP‐selectin level was quantified in patient plasma samples using an ELISA kit (R&D Systems, Minneapolis, MN, USA) according to the manufacturer's instructions.

##### Cell Culture

HepG2 cells, SMC7721 cells, and mouse hepatoma Hep1‐6 cells were obtained from the Cell Resource Center, Chinese Academy of Medical Sciences, Peking Union Medical College. Cells were maintained in Dulbecco's modified Eagle's medium (Hyclone, Logan, UT, USA) containing 10% fetal bovine serum (FBS) (Gibco, Waltham, MA, USA) and incubated at 37 °C in a 5% CO_2_ incubator.

##### Isolation of TAECs

TAECs were obtained from surgical HCC specimens immediately after removal from patients. Specimens were incubated and digested for 1 h at 37 °C in RPMI 1640 medium (Hyclone, Logan, UT, USA) containing 0.1% collagenase IV (Sigma‐Aldrich, St. Louis, MO, USA). The cell suspension was filtrated through a graded series of meshes to separate the cell components from the stroma. The cell suspension was then aggregated after washing in PBS. TAECs were isolated from the cell suspension using magnetic beads coupled with anti‐CD31 monoclonal antibody (Miltenyi Biotech, Bergisch Gladbach, Germany), and magnetic cells were stored with the MACS system (Miltenyi Biotech, Bergisch Gladbach, Germany). To ensure the purity of isolated TAECs, the cell pellets underwent second isolation with anti‐CD31 monoclonal antibodies. Cells were maintained in complete ECM medium (Sciencell, Carlsbad, CA, USA) with 5% FBS, 1% ECGS, 100 U mL^−1^ penicillin, and 100 µg mL^−1^ streptomycin at 37 °C in 5% CO_2_.

##### Transfection Experiments

Lentiviral‐mediated VE‐cadherin‐overexpression vector, ICAM‐1‐overexpression vector, and shRNA ICAM‐1vectors were obtained from GeneChem (Shanghai, China). Cells were seeded at 70–80% confluency in medium and transfected with lentivirus and polybrene A reagent according to the manufacturer's instructions. The empty vector served as a negative control. After 12 h, the medium was removed and replaced with fresh culture medium. Three days later, green fluorescent protein gene expression was observed under a fluorescence microscope, and the cells were collected for subsequent culture.

The siRNAs for Ezrin were chemically synthesized at the Laboratory of RNAChemistry (GeneChem, Shanghai, China) and transfected into TAECs using Lipofectamine 3000 reagent (Invitrogen, Carlsbad, CA, USA) according to the manufacturer's instructions. Target sequences are shown in **Table** **2**. The expression levels of the target genes were analyzed by western blotting.

**Table 2:** The sequences of shRNA for ICAM‐1 and Ezrin and PCR primers

Genes	Sequences	
**shRNA ICAM‐1**		
Control	TTCTCCGAACGTGTCACGT	
shRNA 1	ccGGTATGAGATTGTCATCAT	
shRNA 2	gcCAACCAATGTGCTATTCAA	
shRNA 3	ccAGCCCAAGTTGTTGGGCAT	
**shRNA Ezrin**		
Control	TTCTCCGAACGTGTCACGT	
shRNA 1	ccAGCCAAATACAACAAA	
shRNA 2	ccCACGTCTGAGAATCAACAA	
**qRT‐PCR**		
ICAM‐1	F	TTGGGCATAGAGA CCCCGTT
	R	GCACATTGCTCAGTTCATACACC
VE‐cadherin	F	GTTCACGCATCGGTTGTTCAA
	R	CGCTTCCACCACG ATCTCATA
GAPDH	F	TGTGGGCATCAATGGATTTGG
	R	ACACCATGTATTCCGGGTCAAT

##### Heat Treatment

The plates were sealed with parafilm and submerged in a water bath for 10 min. We previously found that TAECs could not be continuously cultured once the temperature exceeded 47 °C. We selected heat treatments of 37 and 47 °C for 10 min to simulate the control and insufficient RFA treatments, respectively.^[^ ^49^ ^]^

##### TAECs and Platelet Coincubation

Isolated platelets were added into the culture plate or the transwell inserts to coculture with TAECs for 2 h after TAECs were treated with heat treatment, and the plate or transwell insert was then gently washed with PBS three times to remove the platelet.

##### Barrier Model

TAECs (1 × 10^5^) were transferred onto collagen‐coated transwell inserts (length, 12 mm; pore size, 0.4 µm or 8 µm; Corning, Sigma‐Aldrich, USA) in a 24‐well culture dish and incubated at 37 °C for 48–72 h to form a monolayer. Subsequently, TAECs in the barrier model were treated with heat treatment and incubated with platelets to perform in vitro permeability assay and tumor transendothelial assay.^[^ ^50^ ^]^

##### In Vitro Permeability Assay

A fluorescent tracer, 70 KDa (250 µg mL^−1^) tetramethylrhodamine‐dextran (Invitrogen, Carlsbad, CA, USA), was added to the top of the transwell insert, and samples were collected from the lower compartment after 10, 30, 60, and 90 min. The permeability of tetramethylrhodamine‐dextran was detected by measuring the fluorescence intensity of the culture medium in the lower chamber using NOVOstar Microplate Reader (BMG Labtech, Ortenberg, Germany). The permeability of the tracer through the barrier relative to that of untreated controls was calculated.

##### Tumor Transendothelial Assay

HepG2 and SMMC7721 cells (1 × 10^5^) were serum‐starved overnight and labeled with fluorescent cytotracer (Cell Biolabs, San Diego, CA, USA), and they were then added onto inserts. The inserts were transferred to a new plate containing fresh medium with 10% FBS and incubated for 6 h at 37 °C. Non‐migratory HepG2 and SMMC7721 cells were gently removed from the interior membrane of the inserts; cells that migrated to the bottom of the membrane were lysed, and the fluorescence intensity was measured using NOVOstar Microplate Reader.

##### Mice

Male BALB/c nu/nu mice were obtained from Vital River Laboratories (Beijing, China). ICAM‐1‐deficient (ICAM‐1^−/−^) mice were purchased from the Model Animal Research Center of Nanjing University (Nanjing, China) and fully backcrossed to C57BL/6 mice more than 10 generations to transfer into C57BL/6 background. Genotyping of the offspring mice was performed using wild‐type primers (F1, 5ʹ‐CTCATTGTTCAAGGTCTGCCTCAG‐3ʹ; R1, 5ʹ‐ACAGCTCCCCCAAAAG GAAGGA‐3ʹ) and ICAM‐1^−/−^ primers (F2, 5ʹ‐GAGATT CAGTGCCCTCTTCTGGTG‐3ʹ; R2, 5ʹ‐CAGGGTACACCAACCCTGGAATT‐3ʹ). Mice were bred and maintained under specific‐pathogen‐free conditions, and 6‐to‐8‐week‐old littermate mice were used for experiments.

##### Murine HCC Models

Experimental protocols were approved by the Peking University Institutional Animal Care and Use Committee (LA2018088). We generated two murine HCC models, that is, subcutaneous and orthotopic HCC tumor models. In the subcutaneous HCC tumor model, HepG2 cells (5 × 10^6^) in 250 µL serum‐free DMEM were injected subcutaneously into the upper right flank region of nude mice. Mice with established subcutaneous tumors were randomly divided into sham and insufficient RFA groups (*n* = 5 per group). Hep1‐6 cells (5 × 10^6^) were injected into ICAM‐1^−/−^ and wild‐type mice using the same method to establish subcutaneous tumors (*n* = 5 per group). In the orthotopic HCC model, a 30 µL suspension containing HepG2 cells and Matrigel (Corning, Sigma‐Aldrich, USA) was injected into the subcapsular region of the liver parenchyma in the median lobe, as previously described.^[^ ^51^ ^]^ Briefly, 1 × 10^7^ cells in 200 µL of serum‐free DMEM were mixed with Matrigel at a 1:1 (vol/vol) ratio. A 0.5 mL syringe with a 28.5‐gauge needle was used to inject the 30 µL suspension, and the liver surface at the site of the needle tract was covered with Gelfoam for 5 min to minimize bleeding. After four weeks, the tumor reached approximately 1 cm in length for the following experiments. Mice with established orthotopic tumors were randomly divided into sham and insufficient RFA groups (*n* = 7 per group).

##### Sham Procedure and Insufficient RFA In Vivo

Mice were performed with a sham procedure or insufficient RFA when the tumor reached approximately 1 cm in length. In the sham group, a reverse L incision on the abdominal wall was made, and the incision was then sutured. In the insufficient RFA group, a reverse L incision on the abdominal wall was made. To protect adjacent tissues from heat injury, saline cotton balls were placed surrounding the tumor. A radiofrequency current generator (cool‐tip RFA generator, Covidien, Mansfield, MA, USA) was used to generate radiofrequency energy. To deliver the radiofrequency energy, we used a 17‐gauge cool‐tip electrode of 15 cm length with 0.7 cm exposed tip (Covidien, Mansfield, MA, USA). Each ablation cycle lasted for 10 s with a power output of 5 W.

##### In Vivo Assessment of Tumor Metastasis

In the orthotopic HCC model, mice were divided into four groups: 1) Sham + PBS group; 2) Insufficient RFA + C301 (Emfert Analytics, Eibelstadt, Germany) group; 3) Insufficient RFA + R300 (Emfert Analytics, Eibelstadt, Germany) group; and 4) Insufficient RFA + anti‐ICAM‐1 antibody (Biolegend, San Diego, CA, USA) group. One week after insufficient RFA, C301 (2 µg g^−1^ body weight) for isotype control, R300 for platelet deletion (2 µg g^−1^ body weight), or anti‐ICAM‐1 antibody for blocking ICAM‐1 (2 µg g^−1^ body weight) was administered by intraperitoneal injection every three days in the subsequent 34 days. Mice were sacrificed six weeks after insufficient RFA, and livers and lungs were collected and sectioned. The mean volumes and numbers of tumors per lung were calculated. Lungs were fixed in 4% paraformaldehyde (pH 7.5) overnight at 4 °C, dehydrated, and embedded in paraffin. Samples were sectioned at 8‐mm thickness for hematoxylin and eosin (H&E) staining.

##### In Vivo Assessment of Vascular Permeability

Tumors were surgically exposed, and insufficient RFA was performed when the subcutaneous tumors reached approximately 1 cm in length (approximately four weeks after injection). Two weeks later, in vivo multiphoton imaging was performed, as previously described.^[^ ^52^, ^53^ ^]^ Mice were anesthetized with 40 mg kg^−1^ pentobarbital sodium and were then injected with tetramethylrhodamine‐dextran (40 KDa, 0.1 mL of 10 mg mL^−1^, Invitrogen, Carlsbad, CA, USA) via the tail vein. After 5 min, tumors were surgically exposed, and vascular leakage was visualized using multiphoton microscopy. The relative fluorescence intensity across the vessels was analyzed with ImageJ (NIH) software.

##### Western Blotting

Cells were lysed, and protein concentration was determined using a bicinchoninic acid protein assay kit (KeyGEN, Nanjing, China). Protein samples (30 µg) were separated by 8% SDS/PAGE and transferred to a nitrocellulose membrane. Membranes were immersed in 5% non‐fat milk for 1 h and incubated with primary antibody at 4 °C overnight. Primary antibodies were as follows: anti‐VE cadherin (1:1000; ab33168, Abcam, London, UK), anti‐ICAM‐1 (1:1000; 4915S, CST, MA, USA), anti‐Ezrin (1:1000; 3145S, CST, MA, USA), and anti‐*β*‐actin (1:2000; ab8226, Abcam, London, UK). Membranes were then incubated with HRP‐conjugated secondary antibodies at room temperature for 1.5 h. Immunoreactivity was detected with SuperSignal West Pico substrate (Thermo Scientific, Rockford, IL, USA).

##### Immunofluorescence

TAECs cultured to 40–50% confluence on 1 cm culture plates were fixed and blocked, and they were then incubated with primary antibodies including anti‐VE‐cadherin (1:200; ab33168, Abcam, London, UK) and anti‐F‐actin (1:200; ab130935, Abcam, London, UK) overnight at 4 °C. Plates were washed and were then incubated with anti‐mouse or anti‐rabbit fluorescein isothiocyanate‐ and/or tetramethylrhodamine isothiocyanate‐conjugated secondary antibodies (Invitrogen, CA, USA). Cells were counterstained with 4′‐6‐diamidino‐2‐ phenylindole (KeyGen, Nanjing, China) to visualize nuclei and were observed using an inverted fluorescence microscope equipped with an Olympus Qcolor 3 digital camera.

##### Immunoprecipitation

TAECs were lysed in lysis buffer (50 mmol L^−1^ Tris‐Cl [pH 7.4], 150 mmol L^−1^ NaCl, 5% glycerol, 1% NP‐40, 1 mmol L^−1^ EDTA), supplemented with 4 µg mL^−1^ protease inhibitor cocktail and 1 mmol L^−1^ PMSF (Sigma‐Aldrich, St. Louis, MO, USA). The lysate was centrifuged at 13 000*g* for 10 min, and the supernatant was mixed with 2 µL primary antibody and 100 µL protein Agarose beads (Invitrogen, Carlsbad, CA, USA) for 4 h at 4 °C. Primary antibodies were as follows: anti‐ICAM‐1 (10831‐1‐AP, Proteintech, IL, USA) and anti‐Ezrin (3145S, CST, MA, USA). The negative control was incubated with control IgG (corresponding to the primary antibody). Protein A beads were washed in 250 µL NaCl four times, and western blotting was then performed.

##### Realtime PCR

Total RNA was extracted using Trizol reagent (Invitrogen, Carlsbad, CA, USA) according to the manufacturer's instruction. RNA (1–2 µg) was reverse transcribed into cDNA using the SuperScript III First‐Strand Synthesis Kit (Invitrogen, Carlsbad, CA, USA) and oligo‐dT priming according to the manufacturer's instructions. For qPCR, cDNA was amplified using SYBR green PCR master mix (Applied Biosystems, USA) in a Bio‐Rad C1000 Thermal Cycler using the manufacturer's cycling conditions for 40 cycles. The relative RNA expression level was analyzed using the ‐ΔΔCT calculation method and plotted as fold change versus control. Primer sequences used are shown in Table 2.

##### RNA‐Seq Analysis

RNA‐seq transcriptome library was prepared using TruSeq TM RNA sample preparation Kit from Illumina (San Diego, CA, USA) with 1 µg of total RNA. In brief, messenger RNA was isolated according to the polyA selection method by oligo (dT) beads and then fragmented by fragmentation buffer. Double‐stranded cDNA was then synthesized using a SuperScript double‐stranded cDNA synthesis kit (Invitrogen, CA) with random hexamer primers (Illumina). Subsequently, the synthesized cDNA was subjected to end‐repair, phosphorylation, and “A” base addition according to the Illumina's library construction protocol. Libraries were size selected for cDNA target fragments of 200–300 bp on 2% Low Range Ultra Agarose, followed by PCR amplification using Phusion DNA polymerase (NEB) for 15 cycles. After quantification by TBS380, the paired‐end RNA‐seq sequencing library was sequenced with the Illumina HiSeq Xten (2 × 150 bp read length). The raw paired‐end reads were trimmed and quality controlled by SeqPrep (https://github.com/jstjohn/SeqPrep) and Sickle (https://github.com/najoshi/sickle) with default parameters. Clean reads were separately aligned to the reference genome with orientation mode using HIASAT (https://ccb.jhu.edu/software/hisat2/index.shtml) software. The mapped reads of each sample were assembled by StringTie (https://ccb.jhu.edu/software/stringtie/index.shtml=example) using a reference‐based approach. To identify DEGs (differential expression genes) between two different samples, the expression level of each transcript was calculated according to the Transcripts Per Million reads method. RSEM (http://deweylab.biostat.wisc.edu/rsem/) was used to quantify gene abundances. Rstatistical package software DESeq2 (http://bioconductor.org/packages/stats/bioc/DESeq2/) was utilized for differential expression analysis. In addition, functional‐enrichment analyses, including GO and KEGG, were performed to identify which DEGs were significantly enriched in GO term and metabolic pathways compared with the whole‐transcriptome background, with a Bonferroni‐corrected *p*‐value of ≤0.05. GO functional enrichment and KEGG pathway analysis were carried out by Goatools (https://github.com/tanghaibao/Goatools) and KOBAS (http://kobas.cbi.pku.edu.cn/home.do).

##### Scanning Electron Microscopy (SEM)

Platelets (4 × 10^5^) treated with TAECs or heat in suspension were fixed with 2% paraformaldehyde in PBS and layered on poly‐L‐lysine coated coverslips. Platelets were processed for SEM, as described previously.^[^ ^54^ ^]^ Briefly, platelets were incubated with 1% osmium tetroxide for 30 min, rinsed in MQ water, and dehydrated with a graded alcohol series (20–100%) for 5 min. Samples were dried, mounted onto stubs, and coated with gold prior to imaging on a ZEISS Ultra‐55 Scanning Electron Microscope.

##### Platelet Activation Assay

Platelets were incubated with TAECs with different heat treatments for 2 h. Samples were collected and centrifuged at 700 g for 10 min. Platelets were diluted 40 times with PBS. The sample (250 µL) was incubated with 2 µL FITC‐conjugated CD62P‐specific monoclonal antibody (clone #9E1) and isotype control antibody (Clone #11 711) for 20 min at room temperature, and platelets from each sample were then analyzed using a Becton Dickinson FACScan for flow cytometry to measure the platelet surface expression of CD62P.

##### Platelet Adhesion Assay

The isolated platelets (1 × 10^8^ mL^−1^, 50 µL) were incubated with TAECs for 15 min in 96‐well plates at 37 °C and then washed with PBS three times. The plate was fixed with 95% alcohol for 20 min and then blocked with fatty acid‐free bovine serum albumin for 2 h. Subsequently, the plate was treated with anti‐GPIIb‐FITC antibody (1:100; BioLegend) for 2 h at 37 °C and was then washed with PBS three times. The fluorescence intensity was measured.

##### Platelet Aggregation Assay

Platelets (4 × 10^8^ mL^−1^, 1 mL) were incubated with TAECs for 2 h, and the supernatant was collected. The platelet supernatant (100 µL per well) was pipetted into 96‐well plates and placed into a pre‐warmed (37 °C) Microplate Reader. The absorbance was measured to assess aggregation. Aggregation was monitored for 45 cycles with double orbital shaking for 9 s before each cycle according to the manufacturer's instruction.

##### Statistical Analysis

All statistical analyses were carried out using Graphpad Prism Software 8.0 (GraphPad Software Inc., La Jolla, CA, USA). Data were presented as mean ± standard error of mean (SEM). Kolmogorov‐Smirnov test was used to assess normal distribution. For two groups, Student's *t*‐test or nonparametric test was performed for comparison. For more than two groups, one‐way analysis of variance (ANOVA) and post hoc multiplecom‐parison were performed for intergroup comparisons within the same experiment. Kaplan–Meier curves and log‐rank tests were used to compare disease‐free survival in different patient groups. A *p* value of < 0.05 was considered significant.

## Conflict of Interest

The authors declare no conflict of interest.

## Supporting information

Supporting Information
