# Supplementary material for: ICAM‐1 Activates Platelets and Promotes Endothelial Permeability through VE‐Cadherin after Insufficient Radiofrequency Ablation
Source: Adv Sci (Weinh). 2021 Jan 4;8(4):2002228. doi: 10.1002/advs.202002228 (PMC7887603; doi:10.1002/advs.202002228)
Supplement: Supplementary file 1 — Supporting Information [file ADVS-8-2002228-s001.pdf]

WILEY-VCH

© 2020 Wiley-VCH GmbH

ADVANCED SCIENCE
Open Access

Supporting Information

for *Adv. Sci.,* DOI: 10.1002/advs.202002228

ICAM-1 Activates Platelets and Promotes Endothelial Permeability through VE-cadherin after Insufficient Radiofrequency Ablation

Dr. Jian Kong, Dr. Changyu Yao, Dr. Shuying Dong[+], Dr. Shilun Wu, Dr. Yangkai Xu, Dr. Ke Li, Dr. Liang Ji, Dr. Qiang Shen, Dr. Qi Zhang, Dr. Rui Zhan, Dr. Hongtu Cui, Dr. Changping Zhou, Dr. Haigang Niu, Dr. Guoming Li, Dr. Wenbing Sun, and Dr. Lemin Zheng**

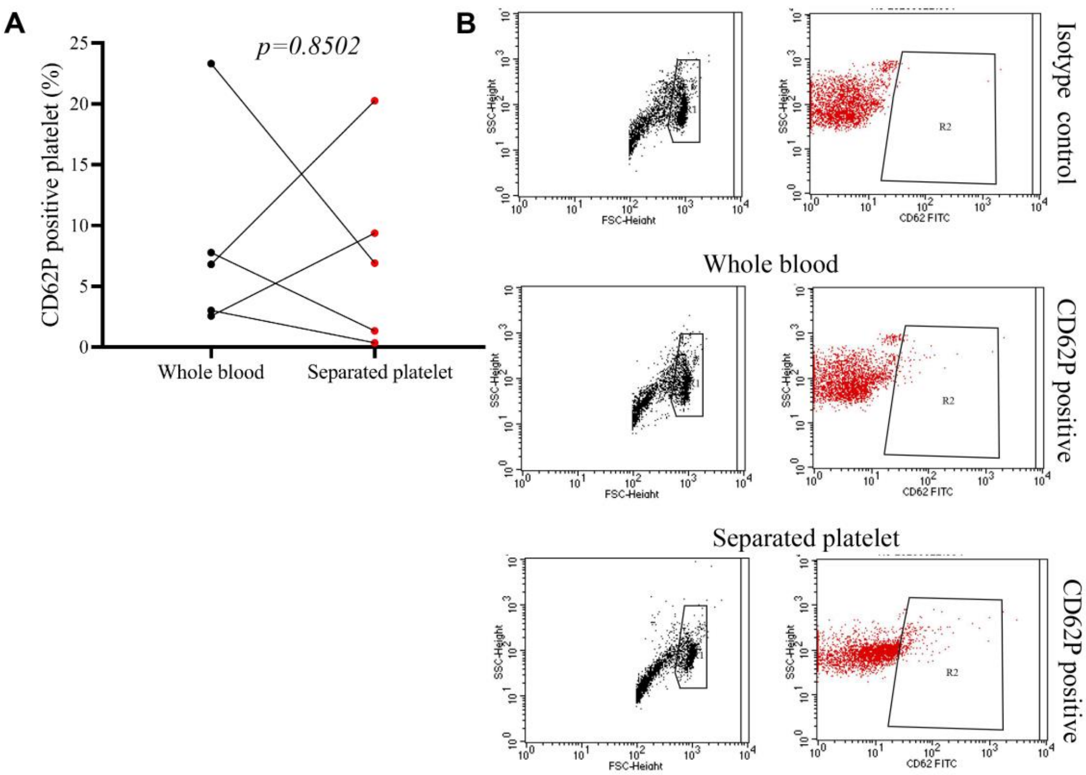

Figure S1. The representative dot plots of flow cytometry. A) The CD62P positive rate of whole blood and separated platelets isolation method (n = 5, P = 0.8502 by paired t-test). B) The representative dot plots of flow cytometry.

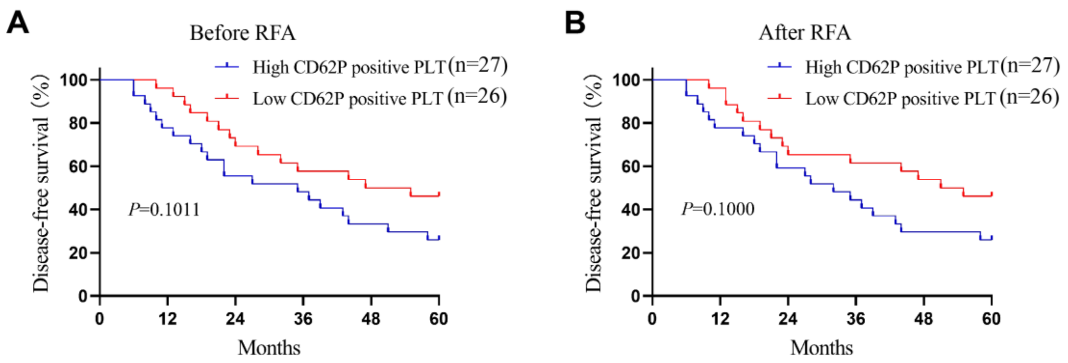

Figure S2. Correlation of CD62P positive platelets with disease free survival in HCC patients. A) High CD62P positive platelet in HCC patients before RFA was correlated with poor disease free survival. B) High CD62P positive platelet in HCC patients after RFA was correlated with poor disease free survival.

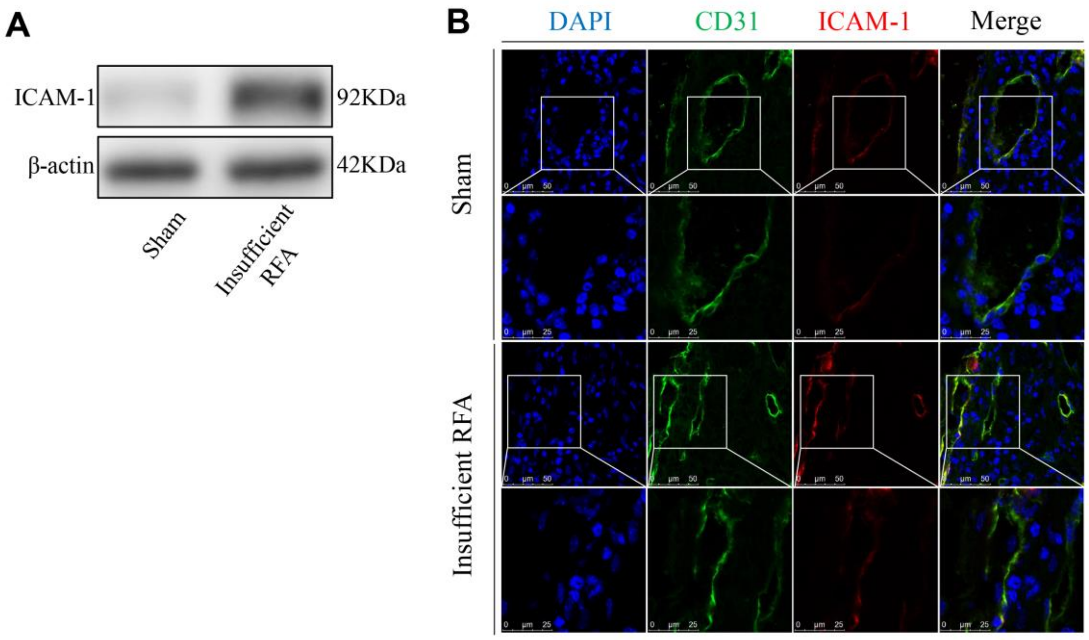

Figure S3. ICAM-1 expression in TAECs from sham operation and insufficient RFA treated mice. A) ICAM-1 expression in TAECs from mice of sham and insufficient RFA group was examined by western blot. B) ICAM-1 in TAECs from mice of sham and insufficient RFA group was examined by laser scanning confocal microscope. Upper: scale bar = 50 µm, Lower: 25 µm.

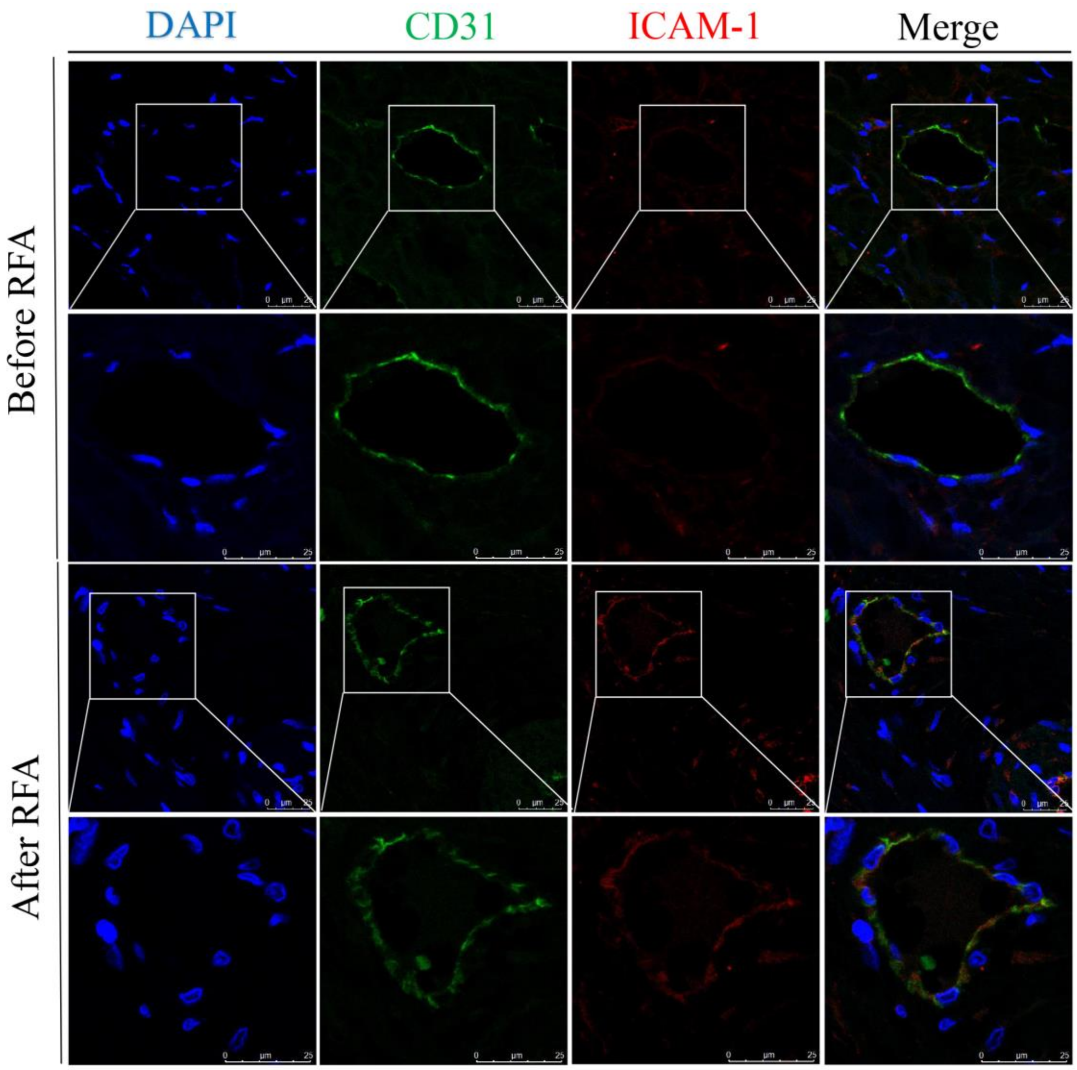

Figure S4. The expression of ICAM-1 in endothelial cells of HCC before and after RFA. ICAM-1 were examined by laser scanning confocal microscope. Scale bar = 25 μm.

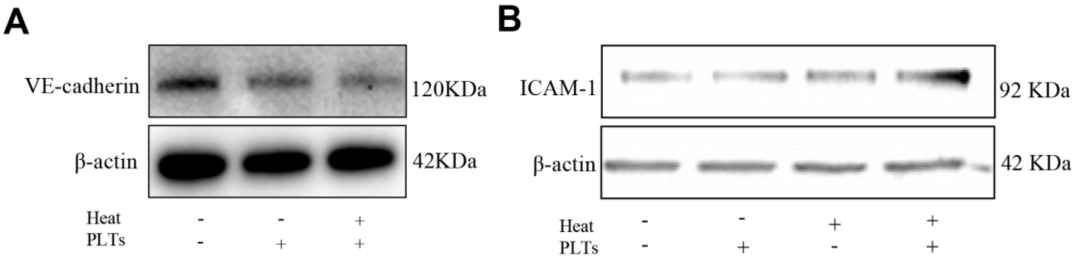

Figure S5. VE-cadherin and ICAM-1 expression in TAECs after heat and/or platelet treatment. A) VE-cadherin expression was examined by western blot after TAECs were treated with heat and platelets. B) ICAM-1 expression was examined by western blot after TAECs were treated with heat and platelets.

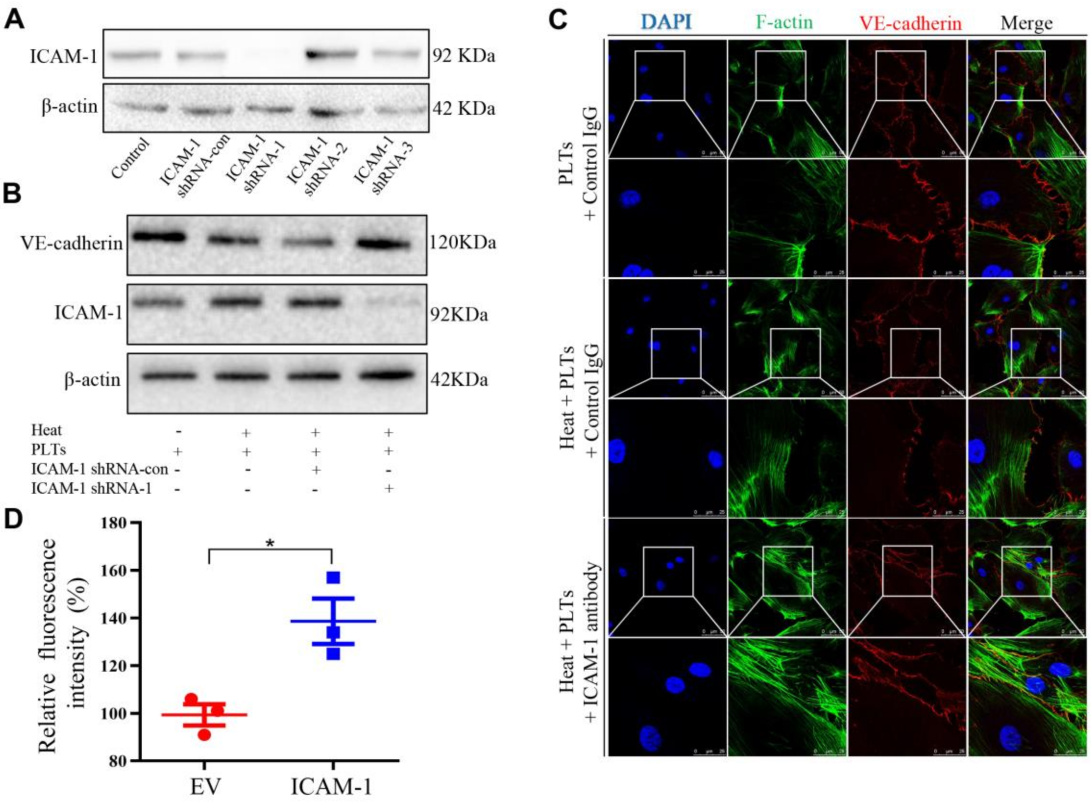

Figure S6. ICAM-1 upregulation in TAECs induces endothelial permeability. A) ICAM-1 expression was knocked down in TAECs using lentivirus and confirmed by western blot. B) The VE-cadherin expression in TAEC was examined by western blot when ICAM-1 shRNA was used to knock down ICAM-1 expression in TAECs with platelet and heat treatment. C) The VE-cadherin expression was examined by laser scanning confocal microscope when ICAM-1 was blocked after TAECs were treated with platelet and heat treatment. Upper: scale bar = 50 μm, Lower: 25 μm. D) Overexpression of ICAM-1 in TAECs enhanced the endothelial permeability (n=3, *P < 0.05 by unpaired t-tset).

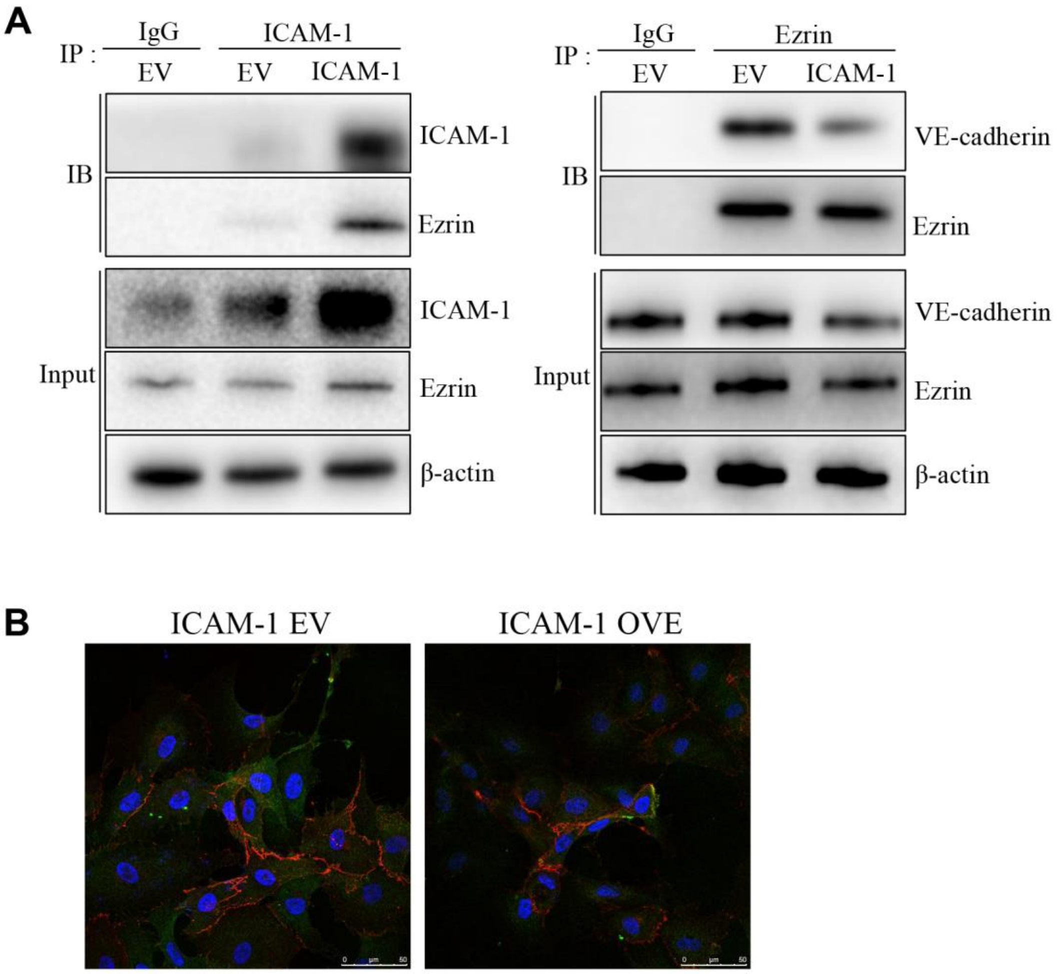

Figure S7. Interaction between ICAM-1, Ezrin and VE-cadherin. A) Whole-cell lysates of ICAM-1 overexpressed TAECs were immunoprecipitated with ICAM-1 and Ezrin respectively, then immunoblotted with antibodies against the indicated proteins. B) Immunofluorescence staining for Ezrin (green), VE-cadherin (red) and DAPI (blue) in ICAM-1 overexpressed TAECs. Scale bar = 50 μm.

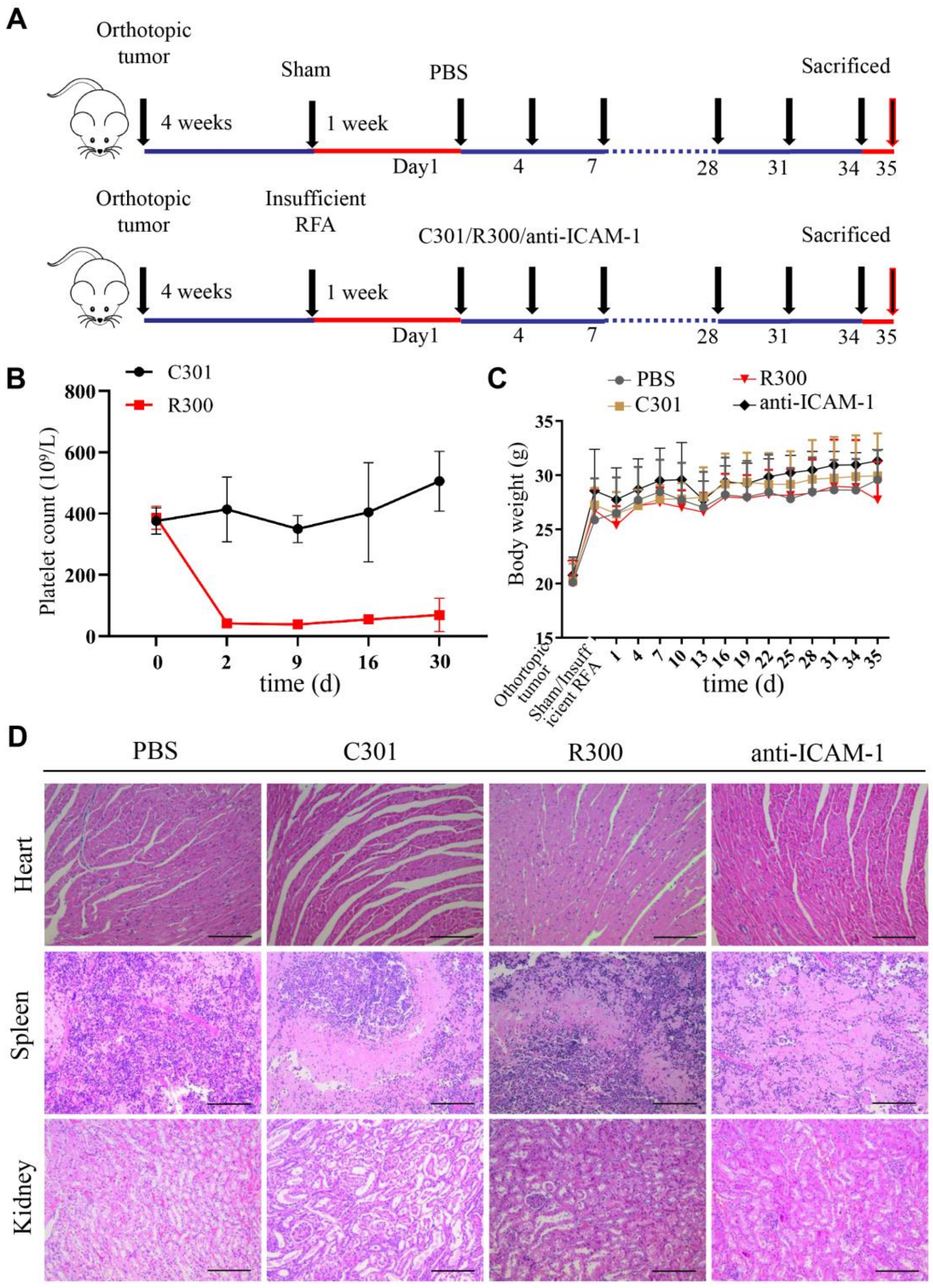

Figure S8. Mice weight and drug toxicity on nude mice bearing with tumor. An orthotopic HCC mouse model was established and insufficient RFA was performed. The mice were treated with PBS, C301 (isotype control of R300), R300 (platelet deletion) and anti-ICAM-1 antibody. A) Schematic showed that the orthotopic HCC

model was established and treated with PBS, C301, R300 or anti-ICAM-1 antibody after sham operation or insufficient RFA. B) The platelet count of mice was assessed during the R300 application. C) Mice weight was measured every 3 days. C) Heart, spleen and kidney sections were stained with H&E. Representative images were displayed. Scale bar = 100 μm.
